# Supplementary material for: Urinary L-FABP as an Early Biomarker for Pediatric Acute Kidney Injury Following Cardiac Surgery with Cardiopulmonary Bypass: A Systematic Review and Meta-Analysis
Source: Int J Mol Sci. 2024 Apr 30;25(9):4912. doi: 10.3390/ijms25094912 (PMC11084509; doi:10.3390/ijms25094912)
Supplement: Supplementary file 1 [file ijms-25-04912-s001.zip › ijms-2969191-supplementary.pdf]

SUPPLEMENTARY MATERIAL:

Supplementary Table S1.

**Database**	**Search equations**	**Results**
Pubmed/MEDLINE	(("Acute Kidney Injury") OR ("Acute Renal Failure")) AND (("Cardiac Surgery") OR ("Cardiopulmonary Bypass") OR (CPB) OR ("Extracorporeal Circulation")) AND (("Liver Fatty Acid Binding Protein") OR ("Liver Fatty Acid-Binding Protein") OR (L-FABP) OR (uL-FABP)) AND ((Urine) OR (Urinary)) AND (Children)	**24**
Web of Science	(("Acute Kidney Injury") OR ("Acute Renal Failure")) AND (("Cardiac Surgery") OR ("Cardiopulmonary Bypass") OR (CPB) OR ("Extracorporeal Circulation")) AND (("Liver Fatty Acid Binding Protein") OR ("Liver Fatty Acid-Binding Protein") OR (L-FABP) OR (uL-FABP)) AND ((Urine) OR (Urinary)) AND (Children)	**28**
Scopus	(("Acute Kidney Injury") OR ("Acute Renal Failure")) AND (("Cardiac Surgery") OR ("Cardiopulmonary Bypass") OR (CPB) OR ("Extracorporeal Circulation")) AND (("Liver Fatty Acid Binding Protein") OR ("Liver Fatty Acid-Binding Protein") OR (L-FABP) OR (uL-FABP)) AND ((Urine) OR (Urinary)) AND (Children)	**508**
Cochrane	(("Acute Kidney Injury") OR ("Acute Renal Failure")) AND (("Cardiac Surgery") OR ("Cardiopulmonary Bypass") OR (CPB) OR ("Extracorporeal Circulation")) AND (("Liver Fatty Acid Binding Protein") OR ("Liver Fatty Acid-Binding Protein") OR (L-FABP) OR (uL-FABP)) AND ((Urine) OR (Urinary)) AND (Children)	**1**

Four databases were searched using main terms such as "Acute Kidney Injury", "Cardiopulmonary Bypass" and "L-FABP" totalizing 561 results prior to the proccess of removal of duplicates.

Supplementary Table S2.

Author	Q1	Q2	Q3	Q4	Q5	Q6	Q7	Q8	Q9	Q10	Overall assessment (Y/T)	Quality assessment
Portilla, 2008	Unclear	Yes	Yes	Yes	NA	Yes	Unclear	Yes	Yes	Yes	7/9 (78%)	GOOD
Krawczeski, 2011	Yes	Yes	No	Unclear	NA	Yes	Unclear	Yes	Yes	Yes	6/9 (67%)	FAIR
Parikh, 2013	Yes	Yes	Yes	Yes	NA	Yes	Unclear	Yes	Yes	Yes	8/9 (89%)	GOOD
Peco-Antić, 2013	Yes	Yes	Yes	Yes	NA	Yes	Yes	Yes	Yes	Yes	9/9 (100%)	GOOD
Zappitelli, 2015	Yes	Yes	Yes	Yes	NA	Yes	Unclear	Yes	Yes	Yes	8/9 (89%)	GOOD
Greenberg, 2018	Yes	Yes	No	Yes	NA	Yes	Unclear	Yes	Yes	No	6/9 (67%)	FAIR
Yoneyama, 2020	Yes	Yes	Yes	Yes	NA	Yes	Unclear	Yes	Yes	Yes	8/9 (89%)	GOOD

Table S2. Quality assessment: JBI's checklist for diagnostic accuracy test studies.

JBI, Joanna Briggs Institute; NA, not applicable; Y/T, yes/total, not applicable were excluded from total; Q1= Was a consecutive or random sample of patients enrolled? Q2= Was a case control design avoided? Q3= Did the study avoid inappropriate exclusions? Q4= Were the index test results interpreted without knowledge of the results of the reference standard? Q5= If a threshold was used, was it pre-specified? Q6= Is the reference standard likely to correctly classify the target condition? Q7= Were the reference standard results interpreted without knowledge of the results of the index test? Q8= Was there an appropriate interval between index test and reference standard? Q9= Did all patients receive the same reference standard? Q10= Were all patients included in the analysis?

Supplementary Table S3.

Author	Q1	Q2	Q3	Q4	Q5	Q6	Q7	Q8	Q9	Q10	Overall assessment (Y/T)	Quality assessment
Ivanisevic, 2013	Yes	Yes	Yes	Yes	Yes	No	No	Yes	Yes	Yes	8/10 (80%)	GOOD
Dong, 2017	Yes	Yes	Yes	Yes	Yes	No	No	Yes	Yes	Yes	8/10 (80%)	GOOD

Table S3. Quality assessment: JBI's checklist for case-control studies.

JBI, Joanna Briggs Institute; NA, not applicable; Y/T, yes/total, not applicable were excluded from total; Q1= Were the groups comparable other than the presence of disease in cases or the absence of disease in controls? Q2= Were cases and controls matched appropriately? Q3= Were the same criteria used for identification of cases and controls? Q4= Was exposure measured in a standard, valid and reliable way? Q5= Was exposure measured in the same way for cases and controls? Q6= Were confounding factors identified? Q7= Were strategies to deal with confounding factors stated? Q8= Were outcomes assessed in a standard, valid and reliable way for cases and controls? Q9= Was the exposure period of interest long enough to be meaningful? Q10= Was appropriate statistical analysis used?
